# DNMT1 interacts with the developmental transcriptional repressor HESX1

**DOI:** 10.1016/j.bbamcr.2007.08.010

**Published:** 2008-01

**Authors:** Ezat Sajedi, Carles Gaston-Massuet, Cynthia L. Andoniadou, Massimo Signore, Paul J. Hurd, Mehul Dattani, Juan Pedro Martinez-Barbera

**Affiliations:** a Neural Development Unit, Institute of Child Health, University College London, 30 Guilford Street, WC1N 1EH, London, UK; b The Wellcome Trust and Cancer Research UK Gurdon Institute, University of Cambridge, Cambridge, UK; c Clinical and Molecular Genetics Unit, Institute of Child Health, University College London, 30 Guilford Street, WC1N 1EH, London, UK

**Keywords:** aa, amino acid, DNMT1, DNA methyltransferase 1, SAFB1, scaffold attachment factor beta 1, RNF2, ring finger protein 2, Lonp2, lon peptidase 2, peroxisomal, ZFP592, zinc finger protein 592, BTBD2, BTB (POZ) domain containing 2, SRFBP1, serum response factor binding protein 1, ZMIZ1, zinc finger MIZ-type containing 1, SOD, septo-optic dysplasia, TLE1, transducin-like enhancer of split 1, N-CoR, nuclear co-repressor, eh1, engrailed homology domain 1, GST, glutathione-*S*-transferase, Gal4DBD, Gal4 DNA binding domain, IVT, in vitro translated, HRP, horse radish peroxidase, PFA, paraformaldehyde, PcP, polycomb group, EZH2, enhancer of zeste homologue, ES cells, embryonic stem cells, DNA methylation, Repression, Homeobox, Forebrain, Pituitary, Mouse

## Abstract

*Hesx1* is a highly conserved homeobox gene present in vertebrates, but absent from invertebrates. Gene targeting experiments in mice have shown that this transcriptional repressor is required for normal forebrain and pituitary development. In humans, mutations in *HESX1* impairing either its repressing activity or DNA binding properties lead to a comparable phenotype to that observed in *Hesx1* deficient mice. In an attempt to gain insights into the molecular function of HESX1, we have performed a yeast two-hybrid screen and identified DNA methyltransferase 1 (DNMT1) as a HESX1 binding protein. We show that *Dnmt1* is co-expressed with *Hesx1* within the anterior forebrain and in the developing Rathke's pouch. Mapping of the interacting regions indicates that the entire HESX1 protein is required to establish binding to a portion of the N-terminus of DNMT1 and its catalytic domain in the C-terminus. The HESX1–DNMT1 complexes can be immunoprecipitated in cells and co-localise in the nucleus. These results establish a link between HESX1 and DNMT1 and suggest a novel mechanism for the repressing properties of HESX1.

## 1 Introduction

*Hesx1* is a transcription factor that belongs to the paired class of homeobox genes. *Hesx1* is conserved in vertebrates, but it is absent from other animal groups, including amphioxus and ascidians. *Hesx1* is expressed in the rostral region of the developing vertebrate embryo, but expression has not been detected in any adult tissues or established cell lines, with the exception of mouse ES cells [1,2]. In mouse, *Hesx1* expression is very dynamic and is regulated by specific enhancers located in the 5′ and 3′ regions of the locus [3]. *Hesx1* transcripts are first detected in the anterior endoderm of the early gastrula embryo, but the most prominent sites of expression are the anterior neural ectoderm, which later gives rise to the forebrain, and Rathke's pouch, the primordium of the anterior pituitary gland [1,2]. The expression pattern of *Hesx1* orthologues in other vertebrates is highly conserved [4,5].

Previous research has shown that *Hesx1* is essential for normal forebrain and pituitary gland formation in mammals [6,7]. *Hesx1*-deficient mouse embryos show variable degrees of forebrain defects, including abnormalities in dorsal midline structures, namely the septum pellucidum, corpus callosum, and anterior and hippocampal commissures. *Hesx1* homozygous mutants also show pituitary dysplasia, anophthalmia or microphthalmia and defects in the olfactory bulbs. A comparable phenotype is observed in the congenital human disorder septo-optic dysplasia (SOD), a syndrome characterised by variable combinations of pituitary abnormalities, midline forebrain defects and optic nerve hypoplasia [6]. Indeed, it has been shown that mutations in human *HESX1* are associated with familial cases of SOD and other forms of hypopituitarism [combined pituitary hormone deficiency (CPHD) and isolated growth hormone deficiency (IGHD)] [8–16].

**Fig. 1:**
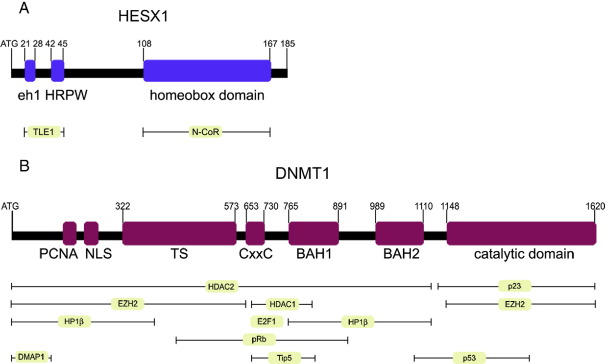
Diagram of HESX1 and DNMT1 proteins showing their functional domains and interacting partners. (A) Two domains in the N-terminal half of HESX1 (eh1 and HRPW) interact with the repressor TLE1, whilst the homeodomain mediates interaction with the N-CoR. (B) DNMT1 contains multiple domains including (1) the PCNA binding motif; (2) the nuclear localisation signal (NLS); (3) the targeting sequence (TS); (4) the CxxC domain; (5) the polybromo domains 1 and 2 (BAH1 and 2); and (6) the catalytic domain. The regions involved in protein–protein interactions with previously reported DNMT1 partners are indicated. This image has been adapted from Spada et al. [21].

At the molecular level, there is evidence indicating that HESX1 can function as a transcriptional repressor in vitro and in vivo [10,11,17]. HESX1 contains two repressor domains, one located in the N-terminus (eh1 and HRPW motifs) and the other in the homeodomain (Fig. 1A). The N-terminal repressing domain binds TLE1, a mammalian orthologue of Groucho, whereas the homeodomain interacts with N-CoR [17]. TLE1 and N-CoR are proteins that mediate transcriptional repression through interactions with DNA binding transcription factors and histone deacetylases [17]. In *Xenopus*, the repressing activity of HESX1 is required for the regulation of both neural differentiation and patterning of the anterior neuroectoderm [18]. Recently, we have shown that the mechanism underlying the forebrain defects in the *Hesx1*-deficient embryos involves the ectopic expression of genes with caudalising activities within the anterior forebrain of the very early mouse embryo [19]. Normal pituitary organogenesis also requires the repressor functions of HESX1 [17]. Therefore, HESX1 is a critical transcriptional repressor that plays a broad role in the development of the forebrain and associated structures, such as the olfactory bulbs, eyes and pituitary gland.

It seems likely that HESX1 exerts its functions not only through the interaction with TLE1 and N-CoR, but further interacting proteins, which remain to be characterised. To search for novel HESX1-interacting proteins, we have performed a yeast two-hybrid screen on a 9.5–10.5 dpc (days post coitum) cDNA mouse library. We have identified DNMT1, a protein responsible for CpG methylation and repression of gene expression, as a HESX1 partner [20–22]. We have mapped the regions of the proteins involved in the interaction and show that HESX1–DNMT1 complexes are present in cells and that both proteins co-localise in the nucleus. We demonstrate, by in situ hybridisation and RT-PCR, that *Dnmt1* is actively transcribed in *Hesx1*-expressing cells in the forebrain and in Rathke's pouch of the developing mouse embryo. In transfected cells, the repressor activity of HESX1, which is mediated by the co-repressors TLE1 and N-CoR, cannot be enhanced by the addition of DNMT1 in a mammalian one-hybrid system. The link between HESX1 and DNMT1 suggests that HESX1 might repress transcription by an alternative mechanism, namely through CpG methylation of HESX1 target genes.

## 2 Experimental procedures

### 2.1 Plasmid constructs

Two bait vectors were constructed by fusing either the full-length HESX1 (aa 1–185) protein or the N-terminal half (aa 1–107, excluding the homeodomain), in frame to the Gal4 DNA binding domain in the pGBDU-C vector [23]. GST–HESX1 and GST–DNMT1 constructs were generated by cloning specific coding sequences into the pGEX-4T vector (Roche). Plasmids containing full-length cDNAs for the interactors were obtained from the IMAGE consortium (MRC Geneservice, Cambridge). These clones were 5706204 (*Dnmt1*), 4021046 (*Rnf2*); 6401542 (*Lonp2*); 5009612 (*Srfbp1*); 6413080 (*Btb2*); 6856060 (*Zmiz1*). An IMAGE clone (6826575) lacking 120 aa of the N-terminal part of *Zfp592* was also obtained. A full-length *Safb1* clone was kindly provided by Dr. Oesterreich (Baylor College of Medicine, USA). Coding regions were amplified by PCR and cloned into the pCMV/SV40-Flag vector (Stratagene) and sequenced. For the mammalian one-hybrid system we used two reporter vectors: (i) p-Gal4BS-SV40 firefly luciferase reporter vector [10,11,17]; (ii) p-P3-SV40 firefly luciferase [17,24]. When cells are transfected with these vectors, expression of luciferase is constitutively active, but it can be repressed by co-transfection of plasmids expressing either Gal4 DNA binding domain (DBD)–HESX1 fusion protein or just full-length HESX1, respectively [10,11,17]. The p-Gal4DBD–Hesx1 construct was built by fusing the full-length coding region of the murine *Hesx1* cDNA to sequences expressing the Gal4DBD in the pM mammalian vector (Clontech). The efficiency of transfection was standardised using the pRL-SV40 renilla luciferase (Promega) as an internal control. Constructs expressing HA–HESX1, Flag–HESX1 and untagged HESX1 were built by cloning the murine *Hesx1* coding sequence into the HA–pCDNA3 (Invitrogen), pCMV/SV40-Flag vector (Invitrogen) and pCAβ-Link [25] vectors, respectively. Primer sequences are available on request.

### 2.2 Yeast two-hybrid screen

We used a 9.5–10.5 dpc mouse expression library, kindly provided by Dr. Weintraub (Howard Hughes Medical Institute, USA) and Dr. Scambler (Institute of Child Health, UK). In this library, cDNAs are fused to a sequence encoding the activation domain of the herpes simplex virus protein VP16 [26]. *Saccharomyces cerevisiae* PJ69-4A strain cultures were independently transformed with the two bait plasmids and single clones were assayed for protein expression by Western blot using a HESX1 antibody [19]. Protein levels were much higher in yeast clones expressing the Gal4DBD–HESX1 (aa 1–107) than in those expressing Gal4DBD–HESX1 (aa 1–185) (Sajedi, E. and Martinez-Barbera, J.P., unpublished data). For the yeast two-hybrid screen, 150 μg of plasmid DNA from the expression library (equivalent to approximately 1.0 × 10^6^ molecules) was transformed into yeast strains expressing either Gal4DBD–HESX1 (aa 1–185) or Gal4DBD–HESX1 (aa 1–107), and plated onto medium lacking leucine, uracil, histidine, adenine and supplemented with X-alpha-Gal and 3-amino triazol as described [26]. After selection, 200 resistant clones were chosen for further analysis. Putative interactors were amplified by PCR with primers flanking the cloning site of the VP16 vector, purified and sequenced. Primer sequences are available on request. Plasmid DNA was purified from yeast cultures using the QIAprep Spin Miniprep kit (Qiagen) after an initial step of lyticase treatment to digest the yeast cell wall.

### 2.3 GST pull-down experiments

Plasmids expressing GST–HESX1 and GST–DNMT1 sequences were used to transform the *Escherichia coli* BL21 strain. Liquid cultures were grown until OD_600_ reached a value of 0.6. Induction of protein expression was achieved by an addition of 0.2 mM IPTG and incubation at 37 °C for 3–5 h. Bacteria were pelleted at 4 °C and resuspended in lysis buffer containing 20 mM Tris–HCl pH 7.5, 200 mM NaCl, 1 mM EDTA, supplemented with protease inhibitor cocktail (Roche), at a ratio of 10 ml of buffer per liter of culture. After sonication, extracts were centrifuged at 12,000×*g* for 30 min at 4 °C, and cleared lysates were incubated with glutathione–sepharose beads (Roche) following manufacturer's instructions. After five washes in lysis buffer, GST fusion proteins bound to the beads were quantified by performing a Bradford assay (Bradford Reagent, SIGMA), aliquoted and kept at − 80 °C in lysis buffer containing 10% glycerol (binding buffer). In vitro translation was performed using the TNT Quick Couple Transcription/Translation kit (Promega) and ^35^S-methionine (Amersham) as recommended by the manufacturer. Around 2 μg of protein (either GST–HESX1, GST-interactor or GST alone) bound to glutathione–sepharose beads was incubated with the ^35^S-methionine-labelled interactor or ES cell protein extracts for 2 h at 4 °C in binding buffer. After three washes in lysis buffer, an equal volume of 2× SDS-PAGE sample buffer was added and samples were denatured at 100 °C for 5 min. Proteins eluted from the beads were subjected to SDS-PAGE, Coomassie stained, dried and exposed to an autoradiographic film.

### 2.4 Expression analysis in mouse embryos

For RT-PCR, total RNA was isolated from the anterior region of the neural plate of 8.5 dpc embryos and the developing Rathke's pouch of 12.5 dpc wild-type mouse embryos using the RNeasy Micro Kit (Qiagen). First strand cDNA synthesis was performed using the Omniscript RT Kit (Qiagen) according to manufacturer's recommendations. Amplification of *Hesx1*, *Dnmt1*, *Safb1*, *Lonp2*, *Zfp592*, *Srfbp1* and *Gapdh* (endogenous control) was performed using specific primers. Primer sequences were Hesx1s, 5′-cccagatcttcccagtgagacttc-3′ and Hesx1as, 5′-gattctgtcttcctctaagtttagc-3′; Dnmt1s, 5′-agttccgtggctacgaggag-3′ and Dnmt1as, 5′-gtctccgtttggcagctggat-3′; Safb1s, 5′-tgcaggagatggaagaggcatc-3′ and Safb1as, 5′-gccgtgctactctgttcaactg-3′; Lonp2s, 5′-atgtcctccgtgagccccatc-3′ and Lonp2as, 5′-aactgcagggacggacatatc-3′; Zfp592s, 5′-aagtcctcagcacagagacg-3′ and Zfp592as, 5′-aagtggcaaggctggaattacag-3′; Srfbp1s, 5′-atggcggctgaccctcttcct-3′ and Srfbp1as, 5′-cccctggtgttcagtgttaacc-3′; Gapdhs, 5′-ttccagtatgactccactcacg-3′ and Gapdhas, 5′-ggatgcagggatgatgttct-3′. In situ hybridisation on whole embryos and paraffin sections was performed as previously described [17,19].

### 2.5 Antibodies

For immunoprecipitation, anti-Flag M2 monoclonal (SIGMA) and anti-HA rat monoclonal antibodies (SIGMA) were used. Immunoblotting was performed using anti-Flag M2-peroxidase conjugate (SIGMA) and anti-HA peroxidase conjugate rat monoclonal (Roche). DNMT1 was detected using an anti-DNMT1 rabbit polyclonal antibody (AbCam). For immunofluorescence experiments anti-Flag rabbit polyclonal (SIGMA) and anti-HA rat monoclonal (SIGMA) primary antibodies were used and Alexa Fluor 488 goat anti-mouse IgG and Alexa Fluor 594 goat anti-rabbit IgG (Molecular Probes) were used as secondary antibodies.

### 2.6 Cell culture and transfections

293T and CHO cells were grown in Dulbecco's modified Eagle's medium (DMEM) (SIGMA), supplemented with 10% fetal calf serum, 2 mM l-glutamine, 100 U/ml penicillin G and 100 μg/ml streptomycin. For immunoprecipitation experiments, cells were co-transfected with 5 μg of each plasmid at 30–40% confluency using the standard calcium phosphate precipitation protocol (SIGMA). For immunofluorescence experiments, cells were cultured to 70% confluency on glass cover slips and transfected using Lipofectamine 2000 (Invitrogen). For the luciferase assays, cells were plated on 12-well tissue culture plates at a density of 1.25–1.5 10^5^ cells per well, and transfected using Lipofectamine (Invitrogen) the following day. Variable amounts of reporter and effector vectors were used in these experiments, but the total concentration of DNA used for transfections was kept constant at 1.0 μg of DNA per well by adding pBlueScript plasmid (Stratagene). CCE ES cells were grown as described [27].

### 2.7 Immunoprecipitation and immunoblotting

Cells were harvested 48 h after transfection and resuspended in lysis buffer containing 1% Triton X-100 for 20 min on ice. Protein extracts were separated from nuclei by centrifugation at 3000×*g* for 10 min at 4 °C and quantified by performing a Bradford assay (Bradford Reagent, SIGMA). Approximately, 0.5–1.0 mg of total protein was incubated with anti-Flag or anti-HA antibodies bound to protein G sepharose beads for 2 h at 4 °C (Roche). After three washes in lysis buffer, immunoprecipitates were resolved by SDS-PAGE and transferred to Immobilon filters (Millipore). Filters were then immunoblotted using HRP-conjugated anti-Flag or anti-HA antibodies. Immunoreactive proteins were visualised using the ECL Detection Reagent System (Amersham).

### 2.8 Indirect immunofluorescence

Two days after transfection, CHO cells were fixed in 4% PFA for 20 min on ice and washed 3 × 5 min in PBT (phosphate-buffered saline containing 0.1% Triton X-100). After blocking in 10% fetal calf serum, cells were incubated with anti-Flag and anti-HA antibodies at 4 °C overnight. Samples were then washed 4 × 5 min in PBT and incubated with secondary antibodies for 1 h at room temperature. After 3 × 5-min washes in PBT, cells were mounted on slides using Vectashield with DAPI (Vector Laboratories). Samples were viewed with a Leica TCS SP Confocal Microscope using an argon–krypton laser and UV10×/0.4 or 40×/0.5 NA dry HC-PLAPO lens (Leica). Images were captured with Leica TCS NT software, composed of 16 sections and four accumulates and processed using ImageJ 1.30v (National Institutes of Health).

## 3 Results

### 3.1 A yeast two-hybrid screen identifies several HESX1-interacting proteins

We carried out two independent yeast two-hybrid screens using bait vectors expressing either the full-length HESX1 protein (aa 1–185) or the N-terminal half (aa 1–107) fused to the Gal4DBD. After selection, surviving clones were isolated and characterised further. Clones containing sequences in frame with the VP16 activation domain corresponded to sequences encoding nine different proteins (Table 1). Yeast clones encoding overlapping sequences of the same putative interactors were repeatedly identified from the two independent screens.

**Fig. 2:**
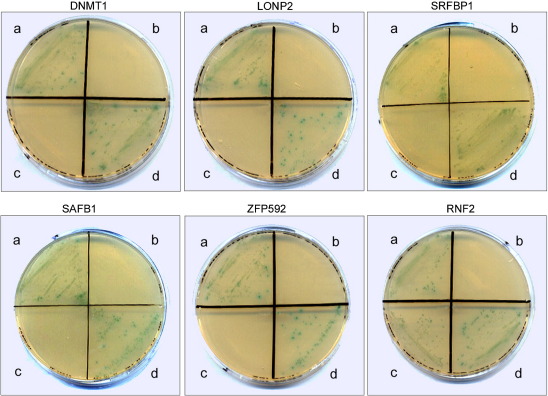
HESX1-interacting proteins in yeast. Transformation of PJ69-4A yeast strain (b), and yeast strains expressing either Gal4DBD–HESX1 (aa 1–185) (a), Gal4DBD–HESX1 (aa 1–107) (d), Gal4DBD alone (c), with plasmids encoding HESX1-interacting proteins fused to VP16 activation domain. Specific interactions are observed with DNMT1, SRFBP1, LONP2, SAFB1 and ZFP592, as shown by the growth of yeasts transformed only with plasmids containing HESX1 sequences (a, d). However, RNF2 is a false positive, as it is able to bind Gal4DBD and to activate the expression of the selecting genes in the absence of HESX1 sequences.

To confirm that the interactors bound to HESX1 sequences, and did not bind Gal4DBD, or activate the expression of the selection markers on their own (auto-activation), we purified plasmids harbouring putative interactor sequences and transformed the PJ69-4A yeast strain and yeast strains expressing either Gal4DBD–HESX1 (aa 1–185), Gal4DBD–HESX1 (aa 1–107) and Gal4DBD. Seven out of nine putative interactors (DNMT1, LONP2, SRFBP1, SAFB1, ZFP592, ZMIZ1 and BTBD2) were able to interact specifically with HESX1 sequences. RNF2 and NRBP2 were found to be false positives (Table 1, Fig. 2 and Sajedi, E. and Martinez-Barbera, J.P., unpublished data).

**Fig. 3:**
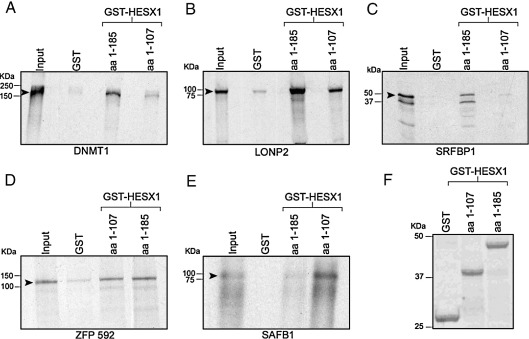
GST pull-down experiments with DNMT1, LONP2, SRFBP1, Zfp592 and SAFB1. HESX1-interacting proteins were in vitro translated in the presence of ^35^S-methionine and incubated with 2.0–3.0 μg of either, GST alone, GST–HESX1 (aa 1–185) or GST–HESX1 (aa 1–107). One tenth of the labelled protein used for the pull-down experiment (arrowheads) was loaded as a control (input). (A–E) Results of GST pull-down experiments with DNMT1 (A), LONP2 (B), SRFBP1 (3), ZFP592 (D) and SAFB1 (E) show specific binding to HESX1 sequences, but only very weakly or not at all to GST. (F) Representative Coomassie stain of the proteins used in the GST pull-down experiments. Molecular weight markers are indicated on the left of each panel. Predicted molecular weights are: 178 kDa (DNMT1); 94 kDa (LONP2); 49 kDa (SRFBP1); 121 kDa (ZFP592); 103 kDa (SAFB1).

To validate these interactions further and to investigate whether they require a direct contact between the proteins, we carried out GST pull-down experiments using GST–HESX1 (aa 1–185), GST–HESX1 (aa 1–107) and GST alone. Three of the interactors, DNMT1, LONP2 and SRFBP1, showed a higher affinity for the GST–HESX1 (aa 1–185) protein when compared with the GST–HESX1 (aa 1–107) (Fig. 3A–C). In contrast, SAFB1 exhibited stronger binding to GST–HESX1 (aa 1–107) (Fig. 3E). In these experiments, ZFP592 binding to both GST–HESX1 fusion proteins was indistinguishable (Fig. 3D).

Among the five identified HESX1 interactors, DNMT1, SAFB1 and SRFBP1 are nuclear proteins that have been previously characterised [20–22,28,29]. ZFP592 is a Kruppel-like C2H2-type zinc finger protein of unknown function [30], whilst LONP2 encodes a Lon peptidase that belongs to the large family of AAA (ATPases associated with diverse cellular activities) proteases.

### 3.2 Expression analysis of the HESX1 partners in mouse embryos

**Fig. 4:**
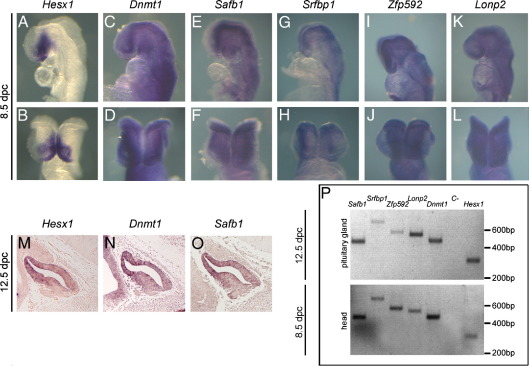
Expression analyses of the HESX1 partners in the forebrain and Rathke's pouch of wild-type mouse embryos. (A–L) Whole-mount in situ hybridisation on wild-type mouse embryos. Probes and developmental stages are indicated on the panels. Top row, lateral view, anterior to the left. Bottom row, rostral view. All interactors are co-expressed with *Hesx1*. (M–O) In situ hybridisation on sections of Rathke's pouch of 12.5 dpc mouse embryos. Note the high-dorsal to low-ventral gradient of expression of *Hesx1*, *Dnmt1* and *Safb1*. (P) RT-PCR from RNA samples purified from mouse pituitaries at 12.5 dpc and mouse heads at 8.5 dpc for *Hesx1* and the five interactors. Amplification of the RNA samples prior to retrotranscription (c-) demonstrates the absence of DNA contamination.

Whole-mount in situ hybridisation on 8.5 dpc wild-type embryos was used to investigate whether the identified interactors were co-expressed with *Hesx1* within the anterior forebrain. At 8.5 dpc, *Hesx1* is expressed in the ventral region of the anterior forebrain including the proximal part of the developing optic stalks (Fig. 4A, B) [1,2]. *Dnmt1*, *Safb1*, *Srfbp1*, *Zfp592* and *Lonp2* were all ubiquitously expressed within the neural tube, including the anterior forebrain (Fig. 4C–L). This expression analysis was also performed at 12.5 dpc by in situ hybridisation on paraffin sections to assess the degree of co-expression between *Hesx1* and the interactors in the developing Rathke's pouch. *Hesx1* shows a characteristic high-dorsal to low-ventral gradient of expression in Rathke's pouch [1,2,17] (Fig. 4M). *Dnmt1* and *Safb1* both showed the same dorso-ventral gradient in Rathke's pouch, indicating that they are co-expressed with *Hesx1* (Fig. 4N, O). *Lonp2* showed a weak and homogeneous pattern of expression in Rathke's pouch, and *Srfbp1* and *Zfp592* mRNA was not detected by slide in situ hybridisation (data not shown).

RT-PCR on RNA samples isolated from the heads (rostral to the mid-hindbrain boundary) of 8.5 dpc embryos and Rathke's pouch of 12.5 dpc wild-type embryos was carried out to complement the in situ hybridisation analysis (Fig. 4P). In heads, *Dnmt1*, *Safb1* and *Zfp592* appeared to be expressed at higher levels than *Srfbp1* and *Lonp2*, although we did not perform quantitative PCR. In Rathke's pouch, expression of *Srfbp1* and *Zfp592* was barely detectable, but *Dnmt1*, *Safb1* and *Lonp2* mRNA was amplified abundantly. Together, these expression analyses suggest that *Hesx1* is co-expressed with the identified interactors either within the anterior forebrain, Rathke's pouch or both.

Protein localisation was not investigated in mouse embryos because the available anti-HESX1 antibodies (either commercially or our own-produced anti-HESX1 sera) do not work in immunocytochemistry assays. However, immunofluorescence on transfected CHO cells using constructs expressing tag-HESX1 and tag interactors revealed nuclear localisation for HESX1 and all interactors [19–22,28,29], except for LONP2, which localises to both the nucleus and the cytoplasm (Sajedi, E. and Martinez-Barbera, J.P., unpublished data).

We have previously demonstrated that HESX1 is a transcriptional repressor, able to recruit TLE1 and N-CoR to artificial promoters to repress transcription [10,11,17]. There is compelling evidence demonstrating the involvement of DNMT1 in CpG methylation and control of gene expression [20–22,31–33]. Therefore, the interaction of HESX1 with DNMT1 suggests that HESX1 might exert part of its repressing activity through the modification of the target gene expression patterns. We decided to characterise further the physical interaction between HESX1 and DNMT1.

### 3.3 Mapping of the HESX1- and DNMT1-interacting regions

**Fig. 5:**
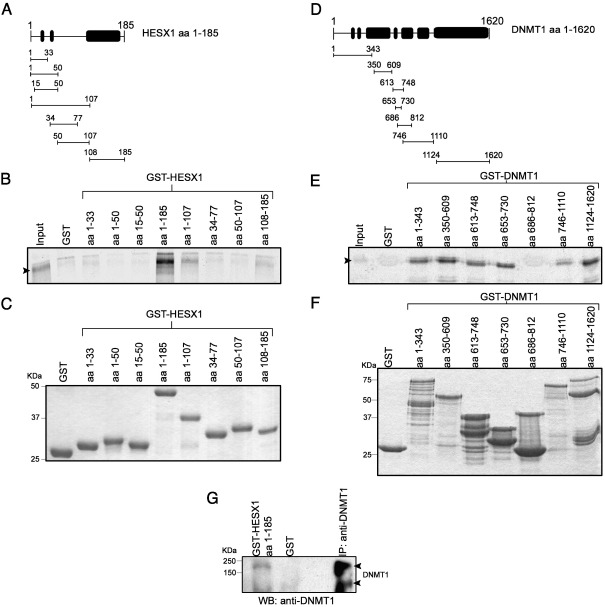
Mapping of the regions responsible for the interaction between HESX1 and DNMT1. (A) Schematic representations of HESX1 fragments expressed as GST fusion proteins. (B) GST pull-down experiments. DNMT1 (aa 1–1620) (arrowhead) strongly interacts with GST–HESX1 (aa 1–185), whilst binding to GST–HESX1 (aa 1–107) is weak. (C) Representative Coomassie stain of the GST–HESX1 fusion proteins used in panel B. (D) Schematic representation of DNMT1 fragments expressed as GST fusion proteins. (E) GST pull-down experiments. Strong interaction with HESX1 (aa 1–185) (arrowhead) is observed with DNMT1 fragments containing the first 748 aa (aa 1–343, aa 350–609, aa 613–7480 and aa 653–730) and the catalytic domain (aa 1124–1620). No binding was detected with a DNMT1 fragment containing the BAH1 domain (aa 686–812). A DNMT1 fragment including BAH1 and BAH2 domains (aa 746–1110) exhibited weak binding. (F) Representative Coomassie stain of the GST–DNMT1 fusion proteins used in panel B. As input, 1/10th of the total amount of ^35^S-Met-labelled protein was used in panels B and E. (G) Approximately 1 mg of protein extracts obtained from untransfected ES cells was incubated with either an anti-DNMT1 antibody (IP: anti-DNMT1), GST or GST–HESX1 (aa 1–185), and bound proteins analysed by Western blot using an anti-DNMT1 antibody. Only GST–HESX1 (aa 1–185), but not GST, interacts with endogenous DNMT1. Molecular weight markers are indicated on the left of each panel.

The GST pull-down assay was used to define the interacting domains on each protein. The use of a series of GST–HESX1 deletion mutant proteins and ^35^S-Met-labelled IVT (in vitro translated) full-length DNMT1 revealed the strongest binding with the full-length HESX1 protein (aa 1–185) (Fig. 5B). Weak binding was observed with the N-terminal part of HESX1 (aa 1–107) and no binding was detected with any of the other HESX1 constructs (Fig. 5B). These data suggest that residues distributed throughout the whole tertiary structure of the HESX1 protein are likely to be required for a complete interaction. However, the N-terminal half of HESX1 confers enough binding properties to form a weak complex in vitro.

GST pull-down assays using ^35^S-Met-labelled IVT full-length HESX1 with a series of GST–DNMT1 mutants encompassing distinct domains were used to map the regions of DNMT1 engaged in the interaction. Binding was consistently observed with peptides containing domains included in the first 748 aa of DNMT1 (Fig. 5E). Numerous functional domains have been mapped to this part of the protein, such as the PCNA binding domain (PBD), which directs association of DNMT1 to the replication foci, the targeting sequence (TS), required for association with peri-centromeric heterochromatin and the cystein-rich region (CxxC) (Fig. 1B) [31–37]. No binding was observed with a deletion protein containing just the bromo homology domain 1 (BAH1), but a weak interaction was supported when both BAH1 and BAH2 were present in the GST–DNMT1 mutant protein (Fig. 5E, aa 686–812 and aa 746–1110, respectively). Residues located in the C-terminal catalytic domain were shown to contribute to the binding interface (Fig. 5E, aa 1124–1620). This is not unexpected, as it has been proposed that the DNMT1 activity depends on the presence of an intra-molecular physical interaction between its N- and C-terminal domains, suggesting that residues from these two regions come in close association in the correctly folded DNMT1 protein [36,37]. HESX1–DNMT1 interaction is mediated by the same regions responsible for the interaction between DNMT1 and the PcG protein EZH2, a histone methyltransferase associated with transcriptional repression [38]. Overall, our data suggest that rather than a specific sequence motif, the tertiary structures of DNMT1 and HESX1 are essential for their interaction.

A GST pull-down assay was also performed on protein extracts obtained from untransfected ES cells, which express *Hesx1* at low levels. GST full-length HESX1 (aa 1–185) was able to bind endogenous DNMT1, as indicated by the detection of an immunoreactive band using a specific anti-DNMT1 antibody in Western blot analysis of GST–HESX1 (aa 1–185) bound proteins (Fig. 5G). No binding was detected to GST, therefore demonstrating a direct interaction to HESX1 and confirming previous results using in vitro translated DNMT1.

### 3.4 HESX1–DNMT1 interaction in mammalian cells

**Fig. 6:**
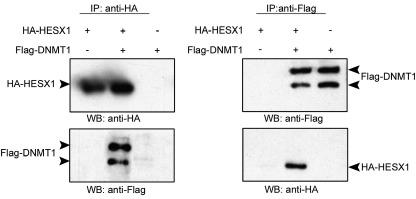
Co-immunoprecipitation of HESX1 and DNMT1 tagged proteins in 293T cells. Cells were transfected with plasmids expressing full-length HA–HESX1 and/or full-length Flag–DNMT1 (indicated by + or −) and immunoprecipitated with anti-flag and anti-HA antibodies. Immunoprecipitates were blotted and detected with anti-HA or anti-Flag HRP-conjugated antibodies. Specific immunoreactive bands are indicated with arrowheads.

To confirm the interaction between HESX1 and DNMT1 further, co-immunoprecipitation of Flag–DNMT1 and HA–HESX1 tagged proteins was performed on transfected cell lines. As shown in Fig. 6, Western blot analysis of the immunoprecipitates with an anti-HA antibody revealed that HESX1 interacts with DNMT1 in 293T cells. Moreover, immunoprecipitation with an anti-Flag antibody followed by Western blot with an anti-HA antibody, also corroborated that HESX1 and DNMT1 form complexes in these cells (Fig. 6). Similar results were obtained in co-immunoprecipitation experiments performed on transfected CHO cells, although protein levels were much lower in this cell line (Sajedi, E. and Martinez-Barbera, J.P., unpublished data).

**Fig. 7:**
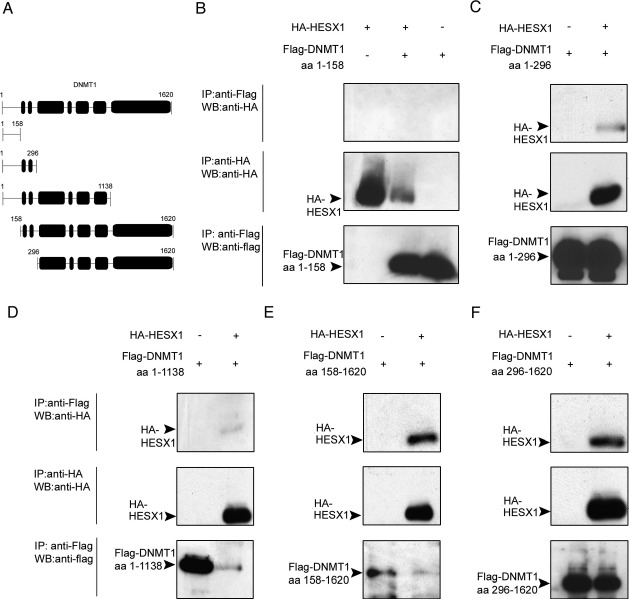
(A) Schematic representation of full-length DNMT1 (aa 1–1620) and fragments used in these experiments. Cells were transfected with full-length HA–HESX1 and/or several Flag–DNMT1 deletion proteins as indicated (+ or −) and immunoprecipitated with anti-Flag or anti-HA antibodies. The presence of HESX1–DNMT1 complexes was analysed by Western blotting the anti-Flag immunoprecipitates with an HRP-conjugated anti-HA antibody. Specific immunoreactive bands are indicated with arrowheads. Fragments containing part of the N-terminus and the catalytic domain of DNMT1 (aa 158–1620 and aa 296–1620) strongly interact with HA–HESX1. Immunoprecipitation with anti-HA antibody was used to verify the presence of HA–HESX1 in the lysates.

We also co-transfected 293T cells with plasmids expressing DNMT1 deletion mutants. These experiments confirmed, as previously described, that the N-terminal half and the C-terminal catalytic domain of DNMT1 are involved in the interaction with HESX1 (Fig. 7). No binding to HESX1 was detected with the DNMT1 fragment (aa 1–158) and weak interaction was observed with a longer peptide encompassing DNMT1 (aa 1–296), which includes the PCNA binding motif (Fig. 7B, C). The DNMT1 catalytic domain (aa 1124–1620) failed to interact with HESX1, but expression levels of this fragment were very low in transfected cells (Sajedi, E. and Martinez-Barbera, J.P., unpublished data).

However, DNMT1 deletion proteins containing the TS and the CxxC domains and the catalytic domain (aa 158–1620 and aa 296–1620) strongly interacted with HESX1 (Fig. 7E, F). Overall, the co-immunoprecipitation experiments suggest that the three-dimensional structure of DNMT1 is required for the interaction.

**Fig. 8:**
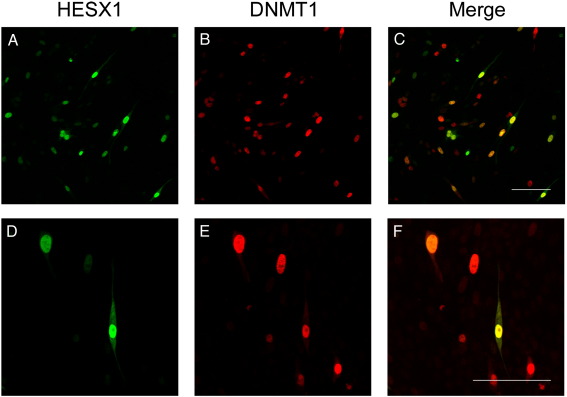
Localisation of HESX1 and DNMT1 in CHO cells. Immunofluorescence was performed on cells transfected with HA–HESX1 and Flag–DNMT1 tagged proteins. (A, D) HA–HESX1 staining (green) is mostly nuclear, but sporadic cells also show cytoplasmic staining. (B, E) Flag–DNMT1 staining (red) is predominantly nuclear. (C) Merged photographs of panels A and B. (F) Merged photographs of panels D and E. Co-localisation of HA–HESX1 and Flag–DNMT1is observed in the nucleus (yellow staining in panels C and F). Scale bar: 100 μm.

Finally, we performed immunocytochemistry to determine the localisation of HESX1 and DNMT1 on co-transfected CHO cells. As shown in Fig. 8, both proteins are predominantly nuclear and were found to co-localise (yellow in merged panels, Fig. 8C, F).

### 3.5 The repressor activity of HESX1 is not enhanced upon binding to DNMT1

**Fig. 9:**
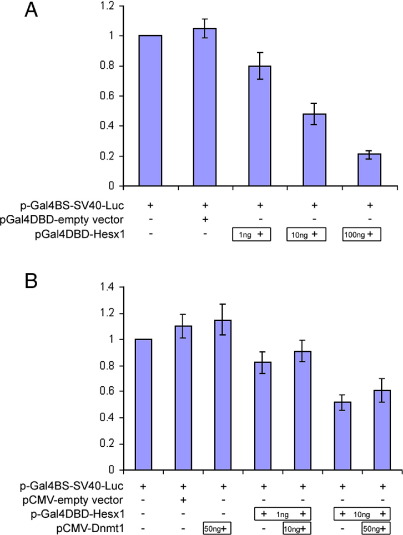
HESX1 repressor activity is not enhanced by DNMT1 in a mammalian one-hybrid system. (A) The basal activation of a luciferase reporter vector containing the SV40 promoter and Gal4 DNA binding sites (p-Gal4BS-SV40-Luc) can be repressed by expression of Gal4 DNA binding domain (DBD)–HESX1 fusion protein from the p-Gal4DBD–Hesx1 effector plasmid in a dose-dependent manner. (B) Co-transfection of DNMT1 cannot increase the repressor activity of the Gal4DBD–Hesx1 fusion protein.

We next examined whether the HESX1–DNMT1 interaction might have an effect on the repressor activity of HESX1. This possibility was investigated using a mammalian one-hybrid system we have previously established in transfected CHO and 293T cells [10,11,17]. In this system, transfection of a construct expressing the HESX1 protein as a fusion to the DNA binding domain (DBD) of Gal4 (Gal4DBD–HESX1) leads to the repression of a constitutively active luciferase reporter (p-Gal4BS-SV40 luciferase) in a dose-dependent manner (Fig. 9A. When cells were co-transfected with a plasmid expressing DNMT1, the repressing activity of Gal4–HESX1 was not increased (Fig. 9B). Similar results to those shown in Fig. 9B were obtained with a range of concentrations of reporter and effector vectors. We also performed luciferase assays using a reporter plasmid containing six copies of the consensus binding site for the paired-like family of homeodomain proteins, which includes HESX1 (p-P3-SV40 luciferase) and effector plasmids expressing the full-length HESX1 protein, rather than a fusion protein to Gal4DBD. In these conditions, addition of DNMT1 also failed to enhance the repressor activity of HESX1 on the P3 reporter vector in a wide range of concentrations (Sajedi, E. and Martinez-Barbera, J.P., unpublished data). Together, these results suggest that binding of DNMT1 to HESX1 cannot increase the repressor activity of HESX1 in transient luciferase assays in transfected cells.

## 4 Discussion

### 4.1 Identification of HESX1-interacting proteins

*Hesx1* is a developmental transcriptional repressor essential for normal forebrain and pituitary formation in mouse and human [6,7]. A total of 13 *HESX1* mutations have been identified so far in association with SOD and/or hypopituitarism: eight of them are point mutations resulting in a single aa change (Q6H, I26T, E149K, R160C, S170L, T181A,Q117P, K176T) [8–12]; one results from the deletion of a guanine in codon 175 (1684delG) causing a frameshift mutation and the generation of a novel C-terminus [13]; a further mutation involves the insertion of an Alu element in the homeodomain leading to skipping of exon 3 (AluIII) [14]; two more result from the insertion or deletion of two nucleotides (306/307insAG and 449/450delAC, respectively) causing a frameshift mutation and the premature termination of translation due to the introduction of a stop codon [15,16]; and a final one is the consequence of a T to C transition at position + 2 of exon 2, which mutates the invariant dinucleotide of the splice donor site of intron 2, yielding a HESX1 truncated protein lacking the homeodomain [16]. The penetrance and severity of the phenotype are highly variable in patients carrying these *HESX1* mutations. At least in part, differences in severity of the phenotypes might be caused by specific perturbations in the protein–protein interactions between particular HESX1 mutated proteins and specific partners. Moreover, the possibility exists for a more direct contribution of HESX1-interacting proteins in human disease, i.e., mutations in HESX1 partners might also lead to congenital hypopituitarism and SOD. These experiments are currently being performed and will be published elsewhere.

To gain insights into the molecular function of HESX1 in normal development and disease, we have carried out a yeast two-hybrid screen and identified five HESX1 interactors. At least three of these are associated with transcriptional repression, whilst the other two are of unknown function. DNMT1 has been shown to play essential roles in several processes including gene silencing. SAFB1 is a transcriptional repressor, which is also involved in chromatin remodelling [39]. SRFBP1 interacts with SRF (serum response factor) to modulate SRF activating properties on a variety of promoters [29].

The expression analysis suggests that all five interactors are co-expressed with *Hesx1* within the anterior forebrain by in situ hybridisation and RT-PCR. Moreover, isolation of pure populations of *Hesx1*-expressing cells from the anterior forebrain and subsequent gene expression profiling has confirmed that all five interactors are co-expressed with *Hesx1* in *Hesx1*-expressing cells (Andoniadou, C.L. and Martinez-Barbera, J.P., unpublished data). *Dnmt1*, *Safb1* and *Lonp2* were highly expressed followed by *Zfp592* and *Srfbp1*, which were weakly expressed. This is consistent with the data obtained from the RT-PCR and in situ hybridisation analysis described here.

Remarkably, we have found that the expression pattern of *Dnmt1* and *Safb1* in the developing Rathke's pouch is very similar to that of *Hesx1*. *Hesx1* expression is high in the dorsal region of Rathke's pouch at 12.5 dpc, where progenitor cells are actively dividing, and low in the ventral part, where cell differentiation is taking place [17]. This suggests that *Dnmt1* and *Safb1* may be required for normal pituitary formation in mouse. In fact, *Safb1*^−/−^ mutant mice die perinatally and surviving individuals exhibit dwarfism due to low levels of insulin-like growth factor 1 [40], a phenotype compatible with hypopituitarism, i.e., isolated growth hormone deficiency. *Dnmt1*^−/−^ mice die early in gestation, but knock-down approaches have revealed that reduction of *Dnmt1* leads to microcephaly (small heads) and defects in terminal differentiation in some organs [31,41]. The pituitary gland was not analysed in these studies.

Together, these findings suggest that HESX1 interacts with several proteins involved in gene repression to execute its function during the development of the forebrain and the pituitary gland.

### 4.2 DNMT1–HESX1 interaction: is there a link between HESX1 and DNA methylation?

In this manuscript, we present evidence supporting the hypothesis that there is a direct interaction between HESX1 and DNMT1, which has been verified (1) in yeast, by a two-hybrid system; (2) in vitro, by GST pull-down assays; and (3) in mammalian cells, by co-immunoprecipitation and co-localisation experiments; (4) by expression analysis in the mouse embryo using RT-PCR and in situ hybridisation. Mapping of the interacting regions suggests that the tertiary structure of both proteins, rather than specific motifs or domains, is required for their interaction. Results obtained from a mammalian one-hybrid system suggest that recruitment of DNMT1 by HESX1 cannot enhance HESX1 activity as a transcriptional repressor. This is in contrast with previous results from our group and from others that have shown that co-transfection of TLE1 or N-CoR, another two known HESX1-interacting proteins, can increase the repressor activity of HESX1 in transfected cells using the same one-hybrid system [10,11,17]. A possibility to explain this discrepancy may be that the interaction of HESX1 with DNMT1 influences the activity of HESX1 in a way that cannot be detected in a one-hybrid system. Alternatively, the functional relevance of the interaction might not be to increase HESX1-repressor activity, but perhaps to silence HESX1 target genes within the *Hesx1*-expressing cells.

Analysis of the *Hesx1*-deficient embryos has shown that the loss of *Hesx1* leads to a posterior transformation of the anterior forebrain [19]. Anterior forebrain structures, such as the eyes and the telencephalic vesicles (which will later form the cerebral hemispheres), are reduced in size, in favour of more posterior structures. At the molecular level, this change of cell fate can be visualised by the ectopic expression of posteriorising genes in the anterior neural ectoderm. The de-repression of posterior marker expression in the anterior forebrain brings about the posterior transformation. This phenotype is consistent with the idea that HESX1 functions as a repressor within the anterior forebrain. Indeed, there is biochemical evidence demonstrating a physical interaction between HESX1 and the co-repressors TLE1 and N-CoR [10,17]. Through these interactions, HESX1 associates in multimeric protein complexes with other co-repressors, such as mSIN3, HDAC1/2 and members of the mammalian SWI/SNF complex, to modify histones, alter chromatin structure and cause repression [17].

Compelling evidence indicates that DNMT1 plays an essential role in the maintenance of transcriptionally repressed chromatin. Methylation of CpG sites is generally inversely correlated with transcriptional activity [20–22]. DNMT1 activity is controlled by its interaction with several proteins, including pRb, DMAP1, HDAC1/2, MeCP2, SUV39H1, HP1beta, p23, p53, EZH2 and the nucleolar chromatin remodelling complex Tip5/Snf2h (Fig. 1) [38,42–50]. These interactions mediate transcriptional repression through independent mechanisms, such as histone deacetylation and methylation, disruption of transcriptional activation complexes and chromatin remodelling. Based on the physical interaction between HESX1 and DNMT1 shown in the present study, we postulate that HESX1 may induce permanent gene silencing by an additional mechanism of action, namely the specific methylation of CpG residues of HESX1 target genes.

One intriguing characteristic of *Hesx1*-deficient embryos is the variable expressivity of the severity of the forebrain and pituitary defects [6,7,19]. This variability was initially thought to be the consequence of a mixed genetic background in the *Hesx1*-deficient embryos. However, *Hesx1* heterozygous mice have been backcrossed with C57BL6/J for more than 20 generations, ensuring a pure genetic background, but variable expressivity of the phenotype still persists [19]. This is not particular to mouse, as similar variability is observed in human patients carrying mutations in *HESX1*, and often between siblings [8–16]. The interaction with DNMT1 can explain the underlying cause of this variable expressivity in the phenotype, as epigenetic modifications have been proven to cause differences in gene expression in identical genotypes [51]. DNMT1-deficient embryos and cells show a generalised genomic hypomethylation and a reduction in gene repression [31,52,53]. It is tempting to speculate that lack of HESX1 in mutant embryos may cause demethylation of specific HESX1 target genes, as has been shown for other DNA binding proteins [38]. The uneven reduction of CpG methylation between mutant embryos of the same genetic background could lead to erratic de-repression and ectopic activation of caudalising genes within the forebrain, and therefore to a variable phenotype. The demonstration of this hypothesis is currently hampered by the lack of knowledge about direct HESX1 targets in vivo. The identification of genes directly regulated by HESX1 will allow us to characterise their methylation patterns in wild-type and *Hesx1*-deficient embryos in vivo, and to correlate these molecular data with the severity of the phenotype of *Hesx1*^−/−^ mutants.

In summary, five HESX1-interacting proteins have been identified in a yeast two-hybrid screen, including DNMT1. The present investigation represents the first step towards the elucidation of the functional consequences of these interactions during development. Future studies will reveal whether the identified HESX1 partners may be involved in the aetiology and pathogenesis of human syndromes, such as SOD and hypopituitarism.

## Figures and Tables

**Table 1:** Results from the yeast two-hybrid screens

HESX1-interacting protein	Accession number	Number of clones^a^	Number of overlapping clones
Scaffold attachment factor β 1 (SAFB1)	XM_128715	20	3
Serum response factor binding protein 1 (SRFBP1)	NM_026040	17	4
DNA methyltransferase 1 (DNMT1)	NM_010066	14	3
Ring finger protein 2 (RNF2)	NM_011277	6	3
BTB (POZ) domain containing 2 (BTBD2)	BC055704	6	1
Lon peptidase 2, peroxisomal (LONP2)	BC049090	3	2
Zinc finger protein 592 (ZFP592)	BC059073	3	2
Zinc finger, MIZ-type containing 1 (ZMIZ1)	BC058646	3	1
Nuclear receptor binding protein 2 (NRBP2)	BC012437	1	1

a SAFB1, SRFBP1, DNMT1, BTB (POZ), ZFP592 and ZMIZ1 were identified in both yeast two-hybrid screens. RNF2 was only identified in the screen using Gal4-DBD-HESX1 (aa 1–107) as bait, whilst LONP2 and NRBP2 were both identified in the screen using Gal4-DBD-HESX1 (aa 1–185) as bait.
